# Survey in radiation oncology departments in Germany, Austria, and Switzerland: state of digitalization by 2023

**DOI:** 10.1007/s00066-023-02182-7

**Published:** 2023-12-05

**Authors:** Stefan Janssen, Rami A. El Shafie, Maximilian Grohmann, Stefan Knippen, Paul M. Putora, Marcus Beck, Andrea Baehr, Patrick Clemens, Sarah Stefanowicz, Dirk Rades, Jan-Niklas Becker, Fabian B. Fahlbusch

**Affiliations:** 1 Department of Radiation Oncology, University of Lübeck, Lübeck, Germany; 2 Private Practice of Radiation Oncology, Hannover, Germany; 3 Clinic of Radiotherapy and Radiation Oncology, University Medical Center Göttingen, Göttingen, Germany; 4 Department of Radiotherapy and Radiation Oncology, University Medical Center Hamburg-Eppendorf, Martinistr. 52, 20246 Hamburg, Germany; 5 Department of Radiation Oncology, Helios Hospitals Schwerin, 19053 Schwerin, Germany; 6 Department of Radiation Oncology, Kantonsspital St. Gallen, St. Gallen, Switzerland; 7 Department of Radiation Oncology, Inselspital, Bern University Hospital and University of Bern, Bern, Switzerland; 8 Department of Radiooncology, Charité – Universitätsmedizin Berlin, corporate member of Freie Universität Berlin and Humboldt Universität zu Berlin, Augustenburger Platz 1, 13353 Berlin, Germany; 9 Department of Radio-Oncology, Academic Teaching Hospital Feldkirch, Carinagasse 47, 6800 Feldkirch, Austria; 10 Department of Radiation Oncology, Klinikum Rechts der Isar, Technical University Munich, Munich, Germany; 11 Department of Radiotherapy and Special Oncology, Medical School Hannover, 30625 Hannover, Germany; 12 Neonatology and Pediatric Intensive Care, Faculty of Medicine, University of Augsburg, 86156 Augsburg, Germany; 13 Department for Human Medicine, MSH Medical School Hamburg, 20457 Hamburg, Germany

**Keywords:** Questionnaires, Information technology, Digital health, Digital workflow

## Abstract

**Purpose:**

The aim of this work was to assess the current state of digitalization in radiation oncology departments in Germany, Austria, and Switzerland.

**Methods:**

A comprehensive survey was conducted in a digital format, consisting of 53 questions that covered various aspects of digitalization including patient workflow, departmental organization, radiotherapy planning, and employee-related aspects.

**Results:**

Overall, 120 forms were eligible for evaluation. Participants were mainly physicians or medical physicists responsible for digitalization aspects in their departments. Nearly 70% of the institutions used electronic patient records, with 50% being completely paperless. However, the use of smartphone apps for electronic patient reported outcomes (ePROMs) and digital health applications (DIGA) was limited (9% and 4.9%, respectively). In total, 70.8% of the radio-oncology departments had interfaces with diagnostic departments, and 36% had digital interchanges with other clinics. Communication with external partners was realized mainly through fax (72%), e‑mails (55%), postal letters (63%), or other digital exchange formats (28%). Almost half of the institutions (49%) had dedicated IT staff for their operations.

**Conclusion:**

To the best of our knowledge, this survey is the first of its kind conducted in German-speaking radiation oncology departments within the medical field. The findings suggest that there is a varied level of digitalization implementation within these departments, with certain areas exhibiting lower rates of digitalization that could benefit from targeted improvement initiatives.

**Supplementary Information:**

The online version of this article (10.1007/s00066-023-02182-7) contains supplementary material, which is available to authorized users.

## Introduction

In recent years, digitalization has gained significant importance in the field of medicine. Radiation oncology, as a naturally innovative specialty, is of particular interest in this regard. According to Aznar et al., the COVID-19 pandemic accelerated the pace of digital transformation processes, further underscoring the significance of digitalization in healthcare [1]. However, less is currently known about the status quo of digitalization in radiation oncology departments. The German Society for Radiation Oncology (DEGRO) established a working group on digitalization in November 2022. This group conducted a survey with the aim of gathering data on the present state of digitalization in radiation oncology departments in German-speaking nations. This survey, which is believed to be the first of its kind, aims to provide evidence of trends and the implementation of various aspects of digitalization. Moreover, the information collected in this publication has importance for optimization and for the future planning and execution of influential projects.

## Methods

To capture the complexity of the term “digitalization,” the survey questions were categorized under different topics (supplementary file 1). These topics ranged from basic information such as country (Germany, Austria, Switzerland), department size, and sponsorship, to more specific areas such as patient workflow, radiotherapy planning, data transfer/interfaces, human resources, media presentation, tumor boards, and digital education.

The survey encompassed a comprehensive range of 53 questions. The selection of questions was made through several consensus meetings with parts of the DEGRO working group for digitalization. The survey included single-choice and multiple-choice questions, free-response questions, and 5‑point Likert scale questions.

The commercially available online survey tool “umfrageonline.com” was utilized for this study. The corresponding survey link was sent to all DEGRO-associated German-speaking institutions across Germany, Austria, and Switzerland (*n* = 372). The head of the department was requested to either complete the survey themselves or to forward it to an employee involved in the topic (e.g., information technology (IT) expert, physician, medical physicist). The survey was available from 10 March to 20 May 2023, and one initial e‑mail and one reminder e‑mail were sent during this period. Participation in the survey was voluntary and anonymous, and all participants provided consent for the publication of the results. Ethical approval was not required for a survey that comprised an anonymous online questionnaire; no patient data were collected.

### Data analysis

The raw data were obtained directly from the online tool “umfrageonline.com” (in Excel Version 16, Microsoft, Redmond, WA, USA) and was subsequently exported to SAS (SAS Institute Inc., Cary, NC, USA) for further analysis. For descriptive analyses absolute and relative frequencies were calculated. Ordinal scales between two subgroups were compared with exact Wilcoxon two-sample tests. Chi-square tests were used to compare nominal data replaced by Fisher’s exact tests, if the expected number of observations in at least one cell was less than 5. A value of *p*< 0.05 was considered statistically significant.

## Results

### General information

The DEGRO office provided 372 e‑mail addresses of radiation oncology institutes across Germany, Austria, and Switzerland. However, 18 of these e‑mail addresses were found to be non-functional, resulting in a total of 354 valid contacts. Of these, 120 institutes participated in the survey, resulting in a response rate of 33.4%. Results about general information are summarized in Table 1.

**Table 1:** Distribution of results concerning general information

Land of origin*	
Germany	92 (76.7%)
Austria	13 (10.8%)
Switzerland	11 (9.2%)
*Participants*	
Physicians	81 (67.5%)
Physicists	22 (18.3%)
IT experts	3 (2.5%)
Others	9 (7.5%)
*Technology interest*	
Very high	73 (60.8%)
High	36 (30%)
Intermediate	9 (7.5%)
small	2 (1.7%)
Institution type**	
University hospital	40 (33.3%)
Non-university hospital	42 (34.9%)
Ambulatory health center	26 (21.7%)
Medical practice	28 (23.4%)
Institution size (therapy units)*	
1	8 (6.7%)
2–3	53 (44.2%)
4–5	29 (24.2%)
6–10	19 (15.8%)
> 10	3 (2.5%)

^*^Differences to 120 (= 100%): not reported; ^**^multiple answers possible

### Workflow

**Fig. 1:**
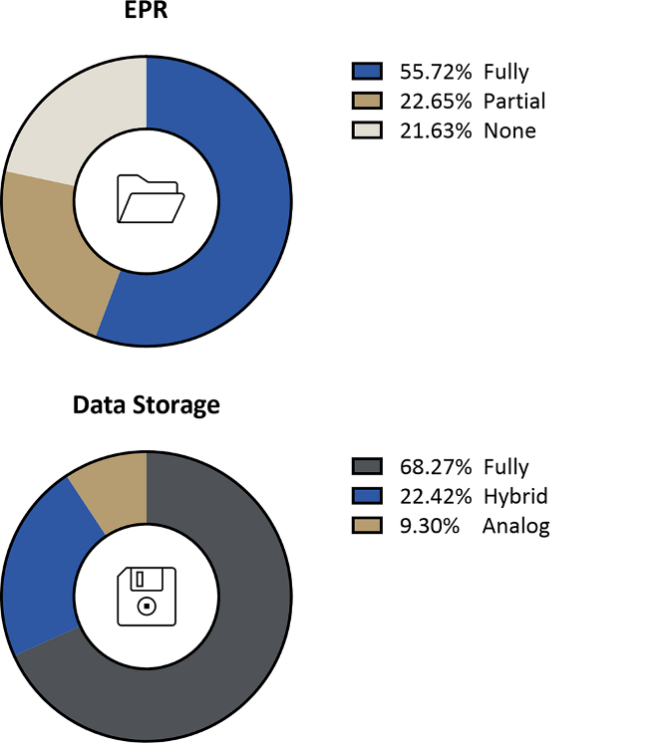
Electronic patients records (EPS) and data storage

**Fig. 2:**
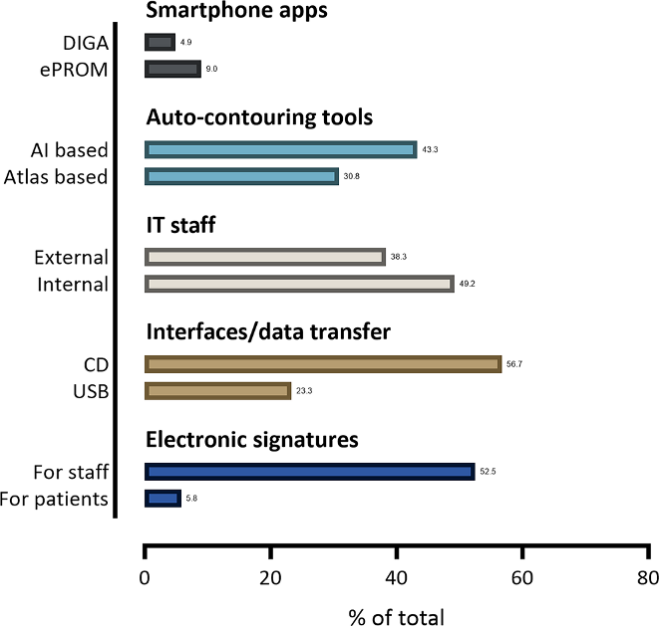
Implementation of various aspects of digitalization in radiation oncology departments. *DIGA* digital health applications, *PROM* electronic patient reported outcome

Overall, 49.2% of the participating institutions had fully implemented electronic patient records. In 20% of the cases, only essential documents were printed out, scanned, and filed separately. In 19.1% of the institutions, no electronic patient record was present, but was mentioned to be implemented in the near future in 13.3% of those cases (Fig. 1). Storage of data was fully digital in one system in 36.7% of the institutions, in different systems in 24.2%, and partially digital (hybrid) in 20% (Fig. 1). No digital storage was present in 8.3%. Most institutions did not use digital appointments (86.7%) or digital appointment reminders (85%). Electronic signatures were applied in 5.8% of cases for patients (e.g., signatures after consultations) and 52.5% for staff (e.g., for RT plan verifications; Fig. 2).

**Fig. 3:**
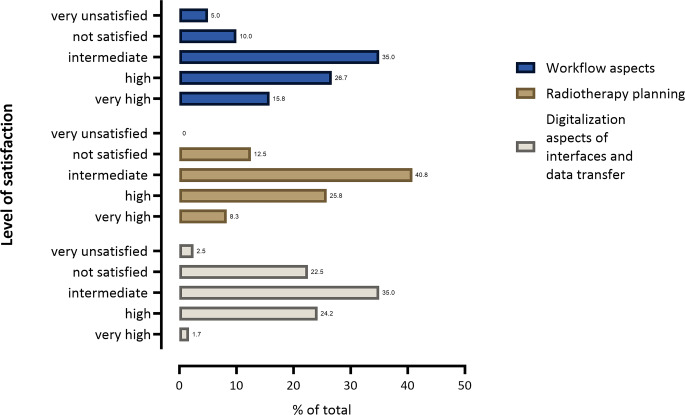
Level of satisfaction regarding workflow, radiotherapy planning, interfaces and data transfer

Automatic patient identification (without staff involvement) was used in 21.6% of departments (e.g., face ID, fingerprint), while manual patient identification was used in 89.2% of cases (e.g., barcode, picture). Nine percent of all institutions used smartphone apps, e.g., for electronic patient reported outcomes (ePROMs), and 4.9% used prescribed smartphone applications (digital health applications, DIGA; Fig. 2). Interestingly, the majority of participants (74.2%) expressed their wish for distributing smartphone apps through a national medical society. In total, 15.8% of all institutions provided the opportunity for online consultation with healthcare professionals, mostly during the follow-up period. Free Wi-Fi for patients was available in 56.7% of the institutions. Satisfaction with the aforementioned workflow aspects was reported as very high, high, and intermediate in 15.8%, 26.7%, and 35% of the cases, respectively, while 10% and 5% were not satisfied or very unsatisfied, respectively (Fig. 3).

### Radiotherapy planning

Auto-contouring tools were implemented in 30.8% (atlas based) and 43.3% (AI based) of cases (Fig. 2). In total, 43.3% of all participants used artificial intelligence (AI)-based adaptive radiotherapy planning. In-house scripting to support the planning process was present in 21.7%, automatic planning tools in 15.5% of cases.

Satisfaction with radiotherapy planning in terms of digitalization aspects was very high, high, and intermediate in 8.3%, 25.8%, and 40.8% of cases, respectively, while 12.5% were not satisfied (0% very unsatisfied; Fig. 3).

### Interfaces/data transfer

**Fig. 4:**
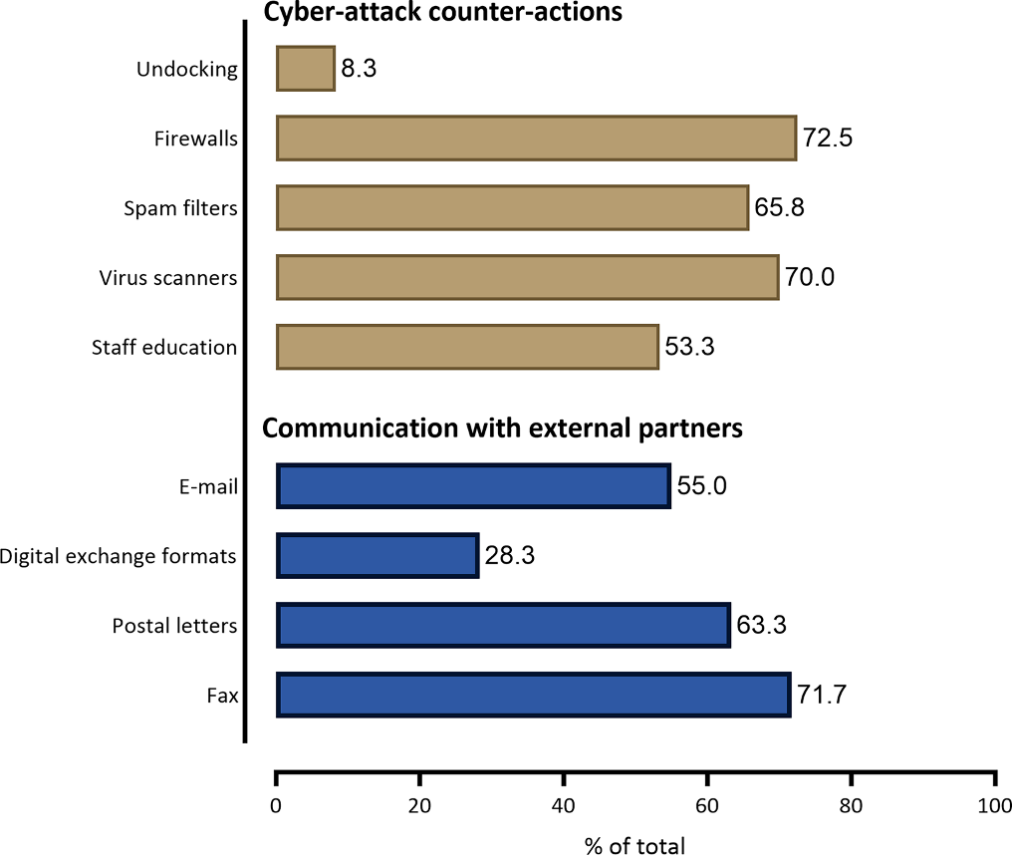
Cyber-attack counter-actions and communication with external partners

During the survey period, the most commonly used radiation oncology information system (ROCIS) was ARIA*®* (56.7%), followed by MOSAIQ*®* (35%). HL7*®* (health level 7) was the most frequently used interface (60.8%), followed by FHIR*®* (fast healthcare interoperability resource, 5%). Most institutions had an interface between ROCIS and the picture archiving and communication system (PACS, 70.8%). Overall, 40% of the institutions had an interface between ROCIS and the hospital information system (H IS), while 42.5% had an interface with the invoice department and 9.5% with the clinical cancer register. Of all the institutions, 35.8% provided the option to transfer data sets to other clinics, e.g., via DICOM*® *(digital imaging and communication in medicine), VPN (virtual private network) tunnel, or cloud-based systems. USB sticks were used in 23.3% of cases to transfer data, while compact disks were used in 56.7% of cases (Fig. 2). Communication with external partners was mainly carried out via fax (71.7%), e‑mails (55%), postal letters (63.3%), and digital exchange formats (28.3%; Fig. 4). Satisfaction with the digitalization aspects of interfaces and data transfer was very high, high, and intermediate in 1.7%, 24.2%, and 35% of cases, respectively, while 22.5% were not satisfied or very unsatisfied (2.5%; Fig. 3).

### IT section

Internal IT staff were responsible for radiation oncology institution issues in 49.2% of participating institutions, while external staff, such as hospital IT experts, were responsible in 38.3% (Fig. 2). The professional background of internal staff varied, with 36.7% being IT specialists, 55% being medical physicists, and 15% being physicians. Servers were locally present in 56.7% of institutions, virtual in 29.2%, and mixed in 31.7%. Most institutions had a blackout concept in place, such as daily backups (69.2%) or geographic separation (45%). Cyberattack counter-actions were present in most institutions, including staff education (53.3%), virus scanners (70%), spam filters (65.8%), firewalls (72.5%), and undocking of internal and external systems (8.3%; Fig. 4).

### Homepage

An institute’s homepage was accessible in 97.5% of cases with 75% being purely descriptive and 9.1% being interactive, allowing for appointments to be arranged or questions to be asked. The institution itself maintained 25% of the homepages, while 25.8% were created by the provider, such as a hospital. A mixture of both was present in 35% of institutions.

### Staff

**Fig. 5:**
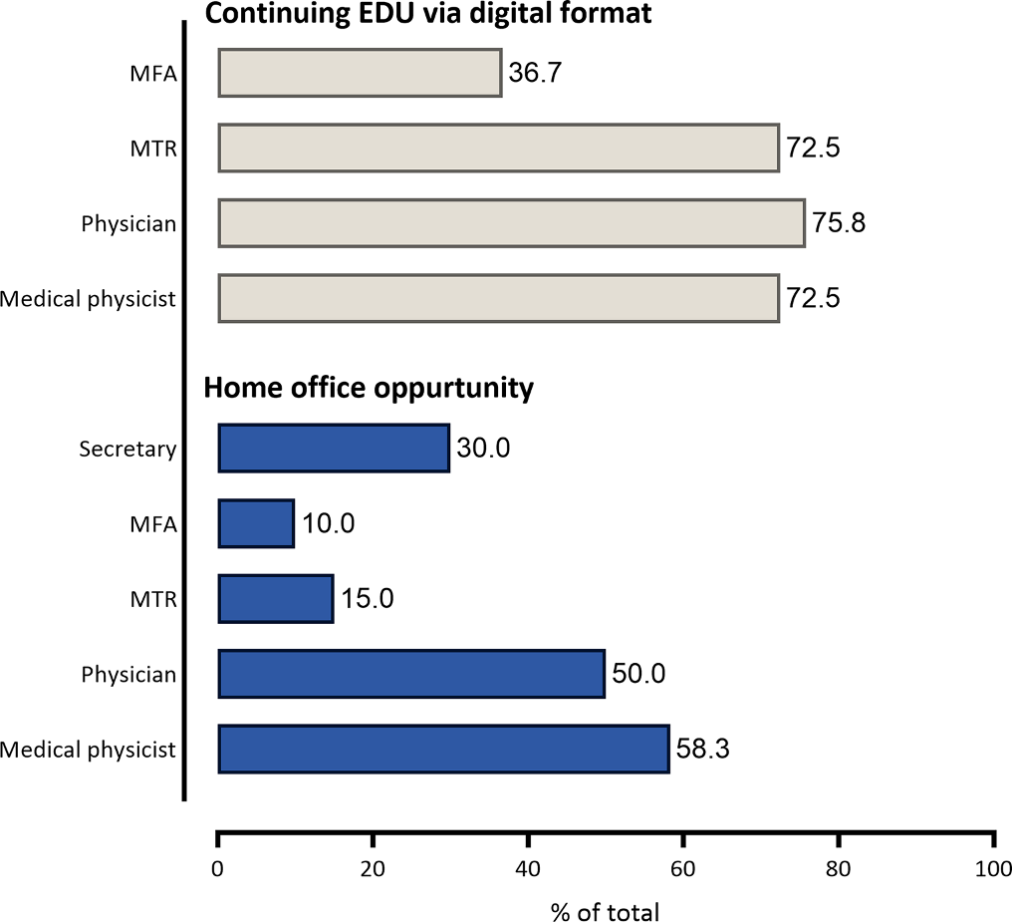
Staff education (EDU) and remonte work opportunities

Home office opportunity was provided for medical physicists (58.3%), physicians (50%), radiographers (MTR)/doctors’ assistants (MFA; 15%/10%), and secretarial staff (30%; Fig. 5). However, in 20% of institutions, there was no chance of working from home. Continuing education in a digital format was offered to physicians (75.8%), medical physicists (72.5%), MTR (72.5%), and MFA (36.7%) staff (Fig. 5). Digital duty rosters were present in 75% of institutions, digital vacation plans in 55%, digital timekeeping in 65.8%, and digital travel management in 28.3%. Educational programs for safe IT usage were offered, with 25.8% being obligatory for new employees and once a year in 17.5% of institutions. Free Wi-Fi for staff was present in 63.3% of institutions.

### Tumor conferences/digital teaching (for university hospitals or teaching hospitals)

Overall, 75% of tumor conferences were regularly conducted digitally (or occasionally, 12.5%). Only 10% of university/teaching hospitals provided digital alternatives for teaching, such as online seminars or literature on servers.

### Future digitalization strategies

Participants nominated several key challenges for digitalization in the future, including mainly data safety (40.8%), compliance (18.3%), data transfer (10.8%), lack of specialists (9.1%) along with legal and financial aspects, interoperability, acceptance, organization, data volume, cybersecurity, documentation, and software performance. More than half of the institutions surveyed (53.3%) had a digitalization strategy in place, and the most commonly cited digitalization projects to be implemented in the next 2 years included digital patient records (15.8%), digital follow-up (12.5%), data transfer (7.5%), AI-based contouring (6.6%), and complete paperless departments (5%). The majority of participants (73.3%) expressed a desire for more comprehensive digitalization.

Significant variations were observed based on the national association of the participants. Participants from Switzerland exhibited a significantly higher percentage of fully paperless patient records (92% vs. 45% in Germany and 36% in Austria, *p* = 0.004), AI adapted auto-contouring (77% vs. 40% in Germany and 46% in Austria, *p* = 0.05), and automatic planning tools (62% vs. 10% in Germany and 18% in Austria, *p* = 0.0001). By contrast, German radiation oncology institutes relied heavily on fax for communication with external colleagues (80% vs. 8% in Switzerland and 13% in Austria, *p* ≤ 0.0001), whereas Swiss institutes preferred e‑mails (100% vs. 50% in Germany and 46% in Austria, *p* = 0.0008).

Moreover, significant variations were also observed between university hospitals, non-university hospitals, and medical practices/ambulatory health centers. In particular, university hospitals showed a notable higher proportion of fully paperless patient records (43% vs. 17% of non-university hospitals and 0% of medical practice, *p* = 0.023) and in-house-scripting solutions (40% vs. 17% of non-university hospitals and 8% of medical practice, *p* = 0.002). Additionally, digital interfaces for data transfer were more present in university hospitals and private practices than in non-university hospitals (45 and 45% vs. 21%, *p* = 0.021).

## Discussion

The Hospital Future Act (*Krankenhauszukunftsgesetz*, KHZG) was passed by the German Bundestag in September 2020 to enhance digital infrastructure in German hospitals. The evaluation process involved the conduction of the so-called DigitalRadar survey to evaluate the digitalization status of German hospitals [2]. In 2022, a preliminary report was released, indicating that 1624 hospitals had an average advancement level of 33.3 out of 100 points. However, it should be noted that the majority of the questions in our survey were specific to radiation oncology departments. As a result, our findings cannot be directly compared to those of the broader DigitalRadar survey, which encompassed a wider range of healthcare departments such as surgical departments and emergency wards. To the best of our knowledge, this is the first survey to evaluate the digitalization status of a specific healthcare specialty.

Electronic health records can improve quality of healthcare [3]. In 2021 Ribelles et al. found electronic health records to be almost universally implemented in medical oncology departments in Spain [4]. In our survey of radiation oncology departments, we found about half of all departments to be fully paperless and another 20% to be partially paperless. Additionally, in 13.3%, this was planned in the near future.

In the context of radiotherapy planning, both in-house scripting solutions and automated contouring and planning tools play pivotal roles in shaping the digitalization of the field. In-house scripting solutions, used by 21.7% of surveyed institutions, offer customization and adaptability, catering to the unique workflows of each department. They provide seamless integration with various software systems and interfaces, adapting as these systems evolve [5, 6]. However, these scripts pose challenges due to their bespoke nature, including the potential for failure and the lack of standardized implementation processes. These factors could hinder their widespread adoption [7, 8]. Furthermore, the relatively low percentage of institutions using in-house scripting might be attributable to a lack of coding skills in radiotherapy departments. Furthermore, 9.1% of the participants expressed concern about the scarcity of specialists, even if coding abilities were present. Additionally, the limited coding access to software products necessitates extensive collaboration with the industry.

Simultaneously, the survey highlights the increasing adoption of automated contouring and planning tools. These tools enhance efficiency, allowing healthcare professionals to focus on patient care. They also improve accuracy and consistency in tasks requiring high precision, leading to more effective treatment plans [9]. AI-based tools add the advantage of adaptability, improving performance with increasing data inputs, and can manage extensive data volumes and complex computations [10, 11]. Despite challenges such as ensuring reliability, data privacy, and seamless workflow integration, these tools represent a significant advancement in radiotherapy.

The use of smartphone applications in the healthcare system has become increasingly common among patients with various diseases. In Germany, the *Digitale-Versorgungs-Gesetz* (DVG) law, which came into effect in 2019, aimed to accelerate the digitalization progress in healthcare [12]. One aspect of this law was the introduction of the possibility to prescribe smartphone apps, known as DIGA. Since then, there has been a steady increase in the prescription of these apps, as reported in the tk-diga report of 2022 [13]. However, in our survey specifically focused on radiation oncology, we found that only 4.8% of institutions were utilizing this option. This low usage could be attributed to the limited number of DIGA applications available in the field of oncology. While the majority of the approximately 50 approved DIGA applications served psychological purposes, only two applications focused on oncology [14]. Furthermore, these two applications were designed for breast cancer patients and not specifically for radiotherapy patients. This fits well with a previous report of the DEGRO working group reviewing apps in the field of radiation oncology [15]. In line with low rates of DIGA application, the utilization of smartphone apps for collecting ePROMs (electronic patient-reported outcomes) was not widespread, possibly due to hesitation in the radiation oncology field where older patients may have limited familiarity with digital technologies. This poses a challenge for app designers to prioritize easy access and user-friendliness, especially for older individuals. However, with the significant increase in smartphone usage in recent years and the projected continuation of this trend, there is an expected future demand for improved accessibility. It is noteworthy that a majority of participants would endorse the provision of medical society-proven smartphone apps (e.g., for collecting ePROMS).

In a survey conducted by the German digital organization Bitkom in 2023, a total of 505 businesses were asked about their internal and external communication methods. The results showed that 82% of the companies still used fax as a means of communication and approximately one third of the companies reported using fax often or very often. However, the frequency of intensive fax usage (40%) had decreased compared to 2018 (62%; [16]). These findings are consistent with our radiation oncology-based research with a general fax usage by 71.7% of all departments, and even 80% of all German departments. It is noteworthy that communication experts have raised concerns about the compliance of fax technology with modern-day European Union data protection laws (*Datenschutz-Grundverordnung, *DSGVO). Furthermore, Austria has implemented national legislation that restricts the use of fax machines to exceptional cases with strict limitations [17]. The prevalence of fax usage may be attributed to the absence of highly secure email accounts among partners. Additionally, radiotherapy facilities in major hospitals frequently rely on their own internal networks, as mandated by linear accelerator manufacturers, resulting in limited connectivity to hospital databases.

Modern image-guided radiotherapy is dependent on IT and data storage applications that are at risk from cyberattacks [18]. In the past 10 years, numerous attacks have led to an interruption of radiation therapy for thousands of patients worldwide [18]. Based on these cases, several recommendations have been published [18–20]. These recommendation range from staff education programs, multifactor authentications, software updates, e‑mail protection filters, and antivirus programs. In line with those recommendations, our survey also revealed a high percentage of daily backups (69.2%), regular staff education (53.3%), virus scanners (70%), spam filters (65.8%), and firewalls (72.5%). Moreover, one key point is having an in-house team with knowledge and understanding of IT related to radiation oncology [19]. In our survey this applied for half of the radiation oncology departments. Still, rating the future challenges in digitalization, data safety and cyberattacks were the most common concerns in our survey.

In recent years, the number of educational resources has increased. A review by Culbert et al. showed e‑learning to be actively integrated into radiation oncology training programs [21]. Even though this study was specifically focused on resident physicians, it aligns with our findings of a significant proportion of continuing education programs being offered in digital format, with rates of 75.8% for physicians and 72.5% for medical physicists.

The results of our survey showed that university hospitals have a higher percentage of paperless patient records and in-house scripting solutions compared to non-university hospitals. Additionally, digital interfaces for data transfer were found to be more common in university hospitals and private practices than in non-university hospitals. These findings suggested that university hospitals may have a more innovative approach than non-university hospitals, which was consistent with a previous analysis of radiotherapy institutions in 2010 [22]. By contrast, however, universities had shortcomings in providing digital alternatives for teaching.

Based on our survey findings, we observed certain discrepancies depending on the national association of the participants. Switzerland, in particular, demonstrated a higher level of innovation, with greater adoption rates of fully paperless patient records, AI-adapted auto-contouring, and automatic planning tools. German radiation oncology departments relied heavily on fax for communication with external colleagues whereas Switzerland radiation oncology departments preferred e‑mails. This fits well with the IMD (International Institute for Management Development) World Digital Competitiveness Ranking, which is published every year. This measures the capacity and readiness of 63 economies to adopt and explore digital technologies as a key driver for economic transformation in business, government, and wider society. Switzerland climbed to the fifth position in the 2022 report, while Germany and Austria remained stagnant at 19th and 18th place, respectively [23]. Although this ranking does not directly reflect the level of digitalization in healthcare, it aligned with our results.

These findings were also in line with a more recent report on healthcare systems by the German Bertelsmann Stiftung, which focused on smart health systems in various European countries, as well as Australia and Canada. The report used 34 indicators to create a digital health index, which encompassed three categories: policy activity, digital health readiness, and actual data utilization. Germany received a digital health score of 30 out of 100, placing it 16th out of 17 countries. Austria and Switzerland achieved scores of 60 and 41, respectively, placing them 10th and 14th [24].

Overall, satisfaction with workflow and automatization was rated positively with only 15% and 12.5% of participants being (very) unsatisfied with those issues.

However, when it came to data transfer, satisfaction levels were lower, with 25% of participants expressing dissatisfaction. This sentiment was echoed in the open-ended responses, where nearly half of the participants identified data transfer/interfaces and data safety as the primary challenges for future digitalization. Furthermore, when it comes to digital data transfer between different institutions, a wide range of diverse solutions were observed. This indicates that there is currently no established standard in place.

In total, approximately 50% of the participating institutions had a specific digitalization strategy planned for the upcoming years. Conversely, a significant majority (73.3%) expressed a strong inclination to further digitalization.

### Limitations

Over the past few years, various surveys on different healthcare topics have been published in the German-speaking radiotherapy community [25–40]. One of the main limitations of these surveys was the low return rate of completed data, which was often correlated with the size of the collective being surveyed. Our survey achieved a higher rollback rate (33.4%) compared to other surveys of similar scale. This is noteworthy considering the larger number of individual questions (*n* = 53) in our survey, compared to a range of 14–42 (median: 26) in the aforementioned surveys.

Nevertheless, the findings of this predominantly descriptive study should be interpreted with caution as the ability to draw correlations was limited due to the relatively small subgroups comprising approximately 30% of all institutes.

Furthermore, it is important to consider that a potential constraint of our research was that over 90% of the participants expressed a significant level of technical interest. This could have introduced a potential bias toward subjective inquiries, such as those pertaining to satisfaction.

## Conclusion and future aspects

Our survey is, to our knowledge, the first of its kind in the medical field, specifically in German-speaking radiation oncology departments, indicating heterogenous implementation of digitalization aspects. This highlights the significance of improving the future coordination of digitalization aspects, which could be accomplished by fostering collaboration among national or international medical societies in radiation oncology. The first steps were already established with building subgroups within the DEGRO working group digitalization (e.g., for data transfer, electronic patient record, artificial intelligence, electronic follow-up) and a cooperation between the DEGRO working group digitalization and the International Society for Radiation Oncology Informatics (ISROI). Within this framework, future objectives can be outlined, ultimately leading to improved benefits both for healthcare professionals and for patients by enhancing coordination and standardizing practices. Our survey identified key challenges in digitalization in radiation oncology, including data safety and interoperability. Future planning should focus on robust cybersecurity measures and the recruitment and training of digitalization specialists. Interoperability issues could be addressed by promoting standardized data formats and protocols. While many institutions had a digitalization strategy, there was a desire for more comprehensive digitalization. Therefore, radiation oncology societies should develop a holistic strategy that includes digital patient records, digital follow-up, data transfer, AI-based contouring, and paperless departments. Collaboration and knowledge sharing should be encouraged through meetings and workshops. Successful strategies and solutions need to be identified and implemented, with a focus on technology adoption. While our survey gives a comprehensive snapshot of the digitalization status it remains crucial to monitor and evaluate further progress through key performance indicators for identifying bottlenecks and areas that require additional support, allowing for timely adjustments and improvements in the transition to digitalization in healthcare. Finally, the adoption of novel technology will require recruitment of human resources and specialized training, involving partnering with technology providers to bridge the skills gap.

### Supplementary Information

original survey questionnaire
