# Supplementary material for: Survey in radiation oncology departments in Germany, Austria, and Switzerland: state of digitalization by 2023
Source: Strahlenther Onkol. 2023 Dec 5;200(6):497–506. doi: 10.1007/s00066-023-02182-7 (PMC11111513; doi:10.1007/s00066-023-02182-7)
Supplement: Supplementary file 1 — original survey questionnaire [file 66_2023_2182_MOESM1_ESM.pdf]

Persönliche Angaben (ausfüllende Person)

Welche Position üben Sie in Ihrer Abteilung aus?

- ◯ Ärztin/Arzt verantwortlich für IT Themen
- ◯ Medizinphysik-Expert:in verantwortlich für IT Themen
- ◯ ausgebildete/r IT Expert:in
- ◯ Andere [ ]

Wie beurteilen Sie Ihr eigenes Technologie Interesse?

[1 sehr groß ▾]

Standort

In welchem Land befindet sich Ihre Institution?

- ◯ Deutschland
- ◯ Österreich
- ◯ Schweiz

Trägerschaft

Welchen Träger hat Ihre Institution?

- ☐ Universitätsklinikum
- ☐ Nicht-Universitäres Krankenhaus (Privater Träger)
- ☐ Nicht-Universitäres Krankenhaus (Kirchlicher Träger)
- ☐ Nicht-Universitäres Krankenhaus (Öffentlicher Träger)
- ☐ Medizinisches Versorgungszentrum (MVZ)
- ☐ Private Praxis (Inhabergeführt)
- ☐ Private Praxis (Investor-getragen)
- ☐ Einzelpraxis
- ☐ Praxis im Praxisverbund
- ☐ Akademisches Lehrkrankenhaus

Abteilungsgröße

Wie viele ... gibt es in Ihrer Abteilung (in Summe bei mehreren Standorten)?

	1	2-3	4-5	6-10	>10
Standorte	○	○	○	○	○
Therapiegeräte	○	○	○	○	○
Fachärzt:innen	○	○	○	○	○
Medizinphysik-Expert:innen	○	○	○	○	○
Weiterbildungsassistent:innen	○	○	○	○	○

Anzahl behandelte Patient:innen/Jahr (in Summe bei mehreren Standorten)

[ ]

Patient:innen/Abteilungs-Workflow

Gibt es in Ihrer Abteilung eine elektronische ("Papierlose") Patientenakte (ePA)?

- ○ Nein
- ○ Nein, aber ist geplant
- ○ Ja, vollständig
- ○ Zum Teil, Hybridlösung (bitte näher ausführen) [ ]

Gibt es in Ihrer Abteilung digitale Patient:innen Unterschriften (beim Aufklärungsbogen)?

- ○ Ja, vollständig
- ○ Ja, in Kombination mit Papierunterschrift
- ○ Nein

Gibt es in Ihrer Abteilung digitale Personal Unterschriften (bei der Planabnahme)?

- ○ Ja, vollständig
- ○ Ja, in Kombination mit Papierunterschrift
- ○ Nein

Gibt es in Ihrer Abteilung digitale Terminvergaben (d.h. Patient:innen können selbst einen Termin vereinbaren z.B. über die Homepage)?

- ○ Nein
- ○ Ja (bitte genauer beschreiben) [ ]

Erhält der/die Patient:in in Ihrer Abteilung digitale Terminerinnerungen?

- ○ Nein
- ○ Ja (bitte genauer beschreiben) [ ]

Welche Formen der AUTOMATISCHEN Patientenidentifizierung (ohne Mitwirkung des Personals) nutzen Sie?

- ◯ Keine
- ◯ Face ID
- ◯ Fingerprint
- ◯ Andere ____

Welche Formen der MANUELLEN Patientenidentifizierung (durch das Personal) nutzen Sie?

- ◯ Keine
- ◯ Barcode
- ◯ Radiofrequenz ID (RFID)
- ◯ Name/Fotokontrolle
- ◯ Andere ____

Nutzen Sie in Ihrer Abteilung eine digitale Archivierung der Patientenakten?

- ◯ Ja, vollständig in einem System
- ◯ Ja, vollständig in mehreren Systemen
- ◯ Ja, zum Teil (Hybrid)
- ◯ Nein

Im Falle einer digitalen Archivierung: Welches Programm nutzen Sie?

____

Nutzen Sie in Ihrer Abteilung Smartphone Apps für Patient:innen (z. B. zur Nachsorge i.S. "patient reported outcomes")?

- ◯ Nein
- ◯ Ja (bitte präzisieren) ____

Würden Sie Apps für Patient:innen in der Nachsorge befürworten (wenn diese beispielsweise von der DEGRO bereitgestellt würden)?

- ◯ ja
- ◯ nein

Nutzen Sie in Ihrer Abteilung "Apps auf Rezept" (Digitale Gesundheitsanwendungen - DIGA)?

- ◯ Nein
- ◯ Ja (bitte präzisieren) ____

Gibt es in Ihrer Abteilung die Möglichkeit für eine online Patientensprechstunde (z.B. über Zoom/Teams/Webex,...)?

- ◯ Nein
- ◯ Ja (Beispiele) ____

Gibt es in Ihrer Abteilung freies WLAN für Patienten:innen?

- ◯ ja
- ◯ nein

Wie zufrieden sind Sie persönlich mit dem digitalen Patient:innen/Abteilungs-Workflow in Ihrer Abteilung?

[1 sehr zufrieden ▾]

Bestrahlungsplanung

Welches Planungssystem nutzen Sie in Ihrer Abteilung?

- ☐ Varian Eclipse
- ☐ Phillips Pinnacle
- ☐ Brainlab Plan
- ☐ RaySearch RayStation
- ☐ Elekta Oncentra Brachy
- ☐ Elekta Monaco
- ☐ Brainlab Elements
- ☐ Accuray Precision
- ☐ Andere ____________

Welche Automatisierungs-Anwendungen im Planungsprozess nutzen Sie in Ihrer Abteilung ?

- ☐ Autokonturierung (Atlanten-basiert)
- ☐ Autokonturierung (KI basiert)
- ☐ KI-basierte adaptive Planung (z.B.: MR-Linac oder Ethos)
- ☐ In-house Scripting im Bestrahlungsplanungs-Prozess
- ☐ Automatische Planerstellungs-Tools (Multi-Criteria-Optimization, Knowledge-based planning, KI)
- ☐ Andere ____________

Wie zufrieden sind Sie persönlich mit der Automatisierung im Bestrahlungsplanungsprozess in Ihrer Abteilung?

[Bitte wählen... ▾]

Schnittstellen/Datentransfer

Welches ROKIS (Radio-Onkologie Klinik Informations-System) nutzen Sie in Ihrer Abteilung?

- ☐ MOSAIQ
- ☐ ARIA
- ☐ RayCare
- ☐ Andere ____________

Welches KIS (Krankenhaus Informations-System/Patientenarchivierungssystem) nutzen Sie in Ihrer Abteilung (z.B. SAP, Orbis, Turbomed, MediStar,...)?

[ ]

Welche Schnittstellen kommen bei Ihnen in der Abteilung zur Anwendung?

- [ ] HL7
- [ ] FHIR
- [ ] Nicht bekannt
- [ ] Andere [ ]

Gibt es in Ihrer Abteilung eine Schnittstelle aus dem ROKIS zum...?

- [ ] KIS
- [ ] Zur Abrechnung
- [ ] zum Klinischen Krebsregister
- [ ] Weiteres [ ]

Im Falle einer Klinik mit angegliedertem MVZ: Gibt es eigene Schnittstellen?

- [ ] Ja (bitte Freitext)
- [ ] Ja, zum Teil (bitte Freitext)
- [ ] Nein
- [ ] [ ]

Gibt es in Ihrer Abteilung eine Schnittstelle zum System der Diagnostischen Radiologie (z.B.: PACS)?

- ○ ja
- ○ nein

Gibt es in Ihrer Abteilung eine Möglichkeit zum digitalen Patientendaten-Austausch mit anderen Kliniken?

- ○ Nein
- ○ Ja (bitte präzisieren) [ ]

Werden in Ihrer Abteilung USB Sticks als Datenträger verwendet?

- ○ ja
- ○ nein

Werden in Ihrer Abteilung CDs als Datenträger verwendet?

- ○ ja
- ○ nein

Wie kommunizieren Sie in Ihrer Abteilung mit externen Zuweisern (z.B.: Befundanfrage)?

- [ ] Fax
- [ ] E-Mail
- [ ] Brief-Post
- [ ] digitale Austauschformate
- [ ] Andere ______

Wie zufrieden sind Sie persönlich mit Datentransfer/den Schnittstellen in Ihrer Abteilung?

Bitte wählen...

IT Abteilung/Server

Wer kümmert sich in Ihrer strahlentherapeutischen Abteilung um IT Belange?

- ( ) externes IT Personal (z.B. Krankenhaus IT)
- ( ) internes Personal (nur für die Strahlentherapie zuständig)

Bei interner Regelung: Wer kümmert sich in Ihrer strahlentherapeutischen Abteilung um IT Belange?

- [ ] ausgebildetes IT-Personal
- [ ] Medizinphysik-Expert:innen
- [ ] Ärzt:innen
- [ ] andere ______

Welche Server gibt es in Ihrer Abteilung?

- [ ] Lokal (zugänglich für Abteilung IT)
- [ ] Virtuell (Cloud-basiert)
- [ ] Gemischt
- [ ] Unbekannt
- [ ] Lokal (z.B. auf dem Klinikgelände, nicht zugänglich für Abteilung IT)

Gibt es ein Ausfallskonzept für einen Serverausfall/zur Datenwiederherstellung?

- [ ] Nein
- [ ] Unbekannt
- [ ] Ja, tägliche Backups
- [ ] Ja, räumliche Brandschutztrennung
- [ ] Ja, sonstiges (z.B. gespiegelte Server, "fail over" Konzept") ______

Gibt es in Ihrer Abteilung Vorkehrungen für einen möglichen "Cyberangriff"?

- [ ] Nein
- [ ] Unbekannt
- [ ] Ja, Personalschulungen zur IT Sicherheit
- [ ] Ja, Virenscanner
- [ ] Ja, Spam Filter
- [ ] Ja, Firewall
- [ ] Ja, sonstiges ______

Homepage

Gibt es für Ihre Abteilung eine eigene Homepage?

- [ ] Nein
- [ ] Ja, rein informativ
- [ ] Ja, mit Interaktionsmöglichkeiten für Patient:innen (bitte präzisieren) ______

Wird die Homepage selbst oder vom Träger gestaltet?

- ○ selbst gestaltet
- ○ vom Träger gestaltet
- ○ teils selbst - teils vom Träger gestaltet

Personal

Für welche der folgenden Berufsgruppen gibt es in Ihrer Abteilung die Möglichkeit einer Homeoffice Tätigkeit (mit digitaler Anbindung zu Patientendaten)?

- [ ] Für Medizinphysik-Expert:innen
- [ ] Für Ärzt:innen
- [ ] Für MTR
- [ ] Für MFA
- [ ] Für Sekretariatsmitarbeiter:innen
- [ ] keine
- [ ] Für Andere ______

Werden in Ihrer Abteilung digitale Fortbildungen genutzt?

- ☐ Für Ärztinnen/Ärzte
- ☐ Für Medizinphysikextert:innen
- ☐ Für MTR
- ☐ Für MFA
- ☐ nein
- ☐ Für Weitere ____________

Gibt es in Ihrer Abteilung ...?

- ☐ digitale Dienstpläne
- ☐ digitale Urlaubsanträge
- ☐ digitale Arbeitszeiterfassung
- ☐ digitale Reisekostenabrechung

Werden Schulungen zum sicheren Umgang mit dem IT System und Patientendaten angeboten?

- ○ Nein
- ○ Ja, verpflichtend (nur bei Einstellung)
- ○ Ja, freiwillig
- ○ Ja, verpflichtend (z.B. jährlich, bitte präzisieren) ____________

Gibt es in Ihrer Abteilung freien WLAN Zugang für Mitarbeiter:innen?

- ○ ja
- ○ nein

Tumorkonferenzen

Finden in Ihrer Abteilung/innerhalb Ihres Tumorzentrums digitale Tumorkonferenzen statt?

- ☐ Ja
- ☐ Nein
- ☐ Bei Bedarf möglich

Digitale Lehre (in Universitätskliniken)

Gibt es in Ihrer Abteilung digitale Lehrangebote im Fachgebiet Strahlentherapie für Student:innen?

- ○ Nein
- ○ Ja (bitte präzisieren) ____________

Abschließende Fragen

Was sind aus Ihrer Sicht die wesentlichen Herausforderungen bei der Digitalisierung in der Strahlentherapie?

Gibt es in Ihrer Abteilung eine Digitalisierungsstrategie für die Zukunft?

- ◯ ja
- ◯ nein

Welche Digitalisierungsprojekte planen Sie in Ihrer Abteilung in den nächsten 1-2 Jahren?

Gibt es in Ihrer Abteilung den Wunsch nach einer stärkeren Digitalisierung?

- ◯ ja
- ◯ nein

Die Umfrage ist beendet.

Vielen Dank für die Teilnahme!

Das Fenster kann nun geschlossen werden.
